# Study on the Mix Design of Mastic Flow for Filling Based on Deviation Coefficient Method

**DOI:** 10.3390/ma19183821

**Published:** 2026-09-08

**Authors:** Yuekai Yao, Min Mi, Kuanghuai Wu, Changkang Lao, Yuqi Zheng

**Affiliations:** 1 Yuexiu Transport Infrastructure Limited, Guangzhou 510030, China; yao.yuekai@yuexiutransport.com (Y.Y.); mi.min@yuexiutransport.com (M.M.); 2 School of Civil Engineering and Transportation, Guangzhou University, Guangzhou 510006, China; lao_ck@163.com (C.L.); zhengyuqi15@163.com (Y.Z.)

**Keywords:** mastic flow for filling (MaFF), deviation coefficient method, mix design, asphalt mixture

## Abstract

To quantitatively evaluate the skeleton design of the MaFF method, this study addresses five dimensions: gradation control, test method, quantitative model, correction conversion, and design method. First, the deviation coefficient method is proposed based on the Talbot method. Unlike conventional design restricted to n = 0.3–0.7, this method takes the maximum density curve at n = 0.45 as the reference and enables flexible adjustment of $V_{ag}$ by controlling the 4.75 mm sieve passing rate. This provides a theoretical basis for discontinuous gradation design. Second, the wet mixing test is developed to overcome aggregate segregation and inadequate lubrication inherent in the dry tamping method, offering a new detection approach that better reflects the actual skeleton structure in the mixture. The optimum parameters were determined as 2% asphalt content, layered loading, one-sided tamping and 75 blows. Using 11 gradations, the wet mixing tests show that $V_{ag}$ increases from 24.13% to 38.29% as λ increases from 0 to 1.0, with a strong linear correlation (R^2^ = 0.996). CT scanning validation indicates that the wet method results are 83–86% of those from 3D reconstruction, yielding a recommended reduction coefficient of 0.84. These findings systematically refine the MaFF method into a complete skeleton design framework. For the specific material system investigated (diabase aggregate, ultra-high-viscosity asphalt, maximum nominal particle size of 13.2 mm), this study provides a preliminary technical reference for FMA mix design. However, broader validation across diverse materials and field conditions is required before generalization.

## 1. Introduction

Asphalt pavement has become the predominant pavement type for highways due to its advantages in driving comfort, construction convenience, and ease of maintenance [1,2]. However, under the long-term effects of heavy traffic loads, temperature variations, and hydrothermal coupling, asphalt pavements frequently suffer from premature distresses such as rutting, cracking, and moisture damage [3,4]. As a result, the actual service life often falls short of the design expectancy [5,6]. These durability challenges have motivated extensive research into advanced mix design methodologies that can systematically improve the mechanical performance and long-term stability of asphalt mixtures.

From a material perspective, asphalt mixture is a multiphase composite material composed of aggregate, filler, and asphalt [7]. Its performance depends not only on the quality of the raw materials but also on the aggregate gradation and the packing characteristics of the skeleton structure. The aggregate skeleton serves as the primary load-bearing framework, and its interlocking behavior is critically influenced by the gradation design. The theoretical foundation of aggregate packing can be traced back to the Fuller–Thompson ideal gradation curve and the Talbot formula, which established the concept of maximum density gradation [7]. Building upon this, the Bailey method introduced a systematic approach for evaluating coarse and fine aggregate interlocking through key sieve ratios [8], while the Superpave method emphasized the control of restricted zones and the use of 0.45-power gradation charts for quality assurance [9]. Despite these advances, conventional design methods—including the Marshall method, Superpave, and Bailey—primarily rely on continuous gradation and maintain design air voids between 3% and 5% to prevent mortar leakage during transportation and paving [10,11]. However, AC mixtures with suspension-dense structures exhibit insufficient high-temperature stability [12], and although SMA mixtures improve rutting resistance through stone-on-stone contact, they remain susceptible to moisture damage due to residual air voids [13]. Haddad and Khedaywi [14] comprehensively reviewed the mechanisms and consequences of aggregate segregation in asphalt concrete mixtures, highlighting that non-uniform particle distribution during handling and placement significantly compromises both the mechanical properties and the long-term durability. These persistent issues indicate that conventional gradation design and compaction simulation approaches have inherent limitations in balancing multiple performance requirements.

In recent years, researchers have explored various approaches to enhance the performance of asphalt mixtures through material modification and gradation optimization. Khedaywi et al. [15] systematically investigated the effect of waste iron powder on the properties of asphalt concrete mixtures, demonstrating that incorporating industrial by-products can improve certain mechanical characteristics while addressing environmental concerns. Similarly, the modification of asphalt binders and mixtures with waste polyethylene has been extensively studied, revealing that polymer modification can enhance rutting resistance and low-temperature cracking performance [16]. The role of filler materials has also received considerable attention. Khedaywi et al. [17] examined the effects of different filler types on the Marshall properties and rutting resistance, while other studies have characterized the performance of asphalt mixtures modified with waste toner, further expanding the range of sustainable modifiers [18]. These findings collectively underscore the importance of systematic mix design and material characterization in achieving durable pavement performance. Furthermore, the void characteristics of asphalt mixtures—particularly voids in the mineral aggregate (VMA) and voids filled with asphalt (VFA)—have been identified as critical parameters governing durability and moisture susceptibility [19]. Recent advances in X-ray CT scanning have enabled non-destructive three-dimensional reconstruction of internal void structures, providing new insights into the spatial distribution and connectivity of voids within asphalt mixtures [20,21]. This technique offers a promising tool for validating laboratory packing measurements against the actual void characteristics of compacted mixtures.

To address the above issues, Wu et al. proposed the fluid mastic asphalt (FMA) mixture [22]. As a novel asphalt mixture, FMA fundamentally resolves the performance contradictions inherent in conventional mixtures, with the goal of achieving long-term durability. The mixture is designed using the Mastic Flow for Filling (MaFF) method, which divides the mix design into two components: skeleton design and mastic design [23,24]. The skeleton is designed to carry vehicle loads and provide high-temperature durability. Chu et al. conducted long-term rutting tests on FMA-13 (6.0) and reported that its high-temperature stability under sustained loading significantly outperforms that of AC-13, with an improvement of several times [25]. During the molding process, the fluid mastic can fully fill the voids in the aggregate, effectively preventing air and moisture from penetrating the mixture, thereby achieving the durability design. Nie et al. demonstrated through direct tensile tests that FMA-13 exhibits superior tensile performance compared to AC-13 and SMA-13 [26]. Zheng et al. further confirmed its advantages in crack resistance, fatigue performance, and self-healing capability using semi-circular bending and four-point bending tests [27,28]. These performance benefits are primarily attributable to the mastic flow-filling design concept, which allows the asphalt–aggregate ratio to exceed conventional limits and enables a wide-range asphalt content design. The resulting higher asphalt content and lower air voids significantly enhance the mixture’s stress absorption capacity. Under the fluid mastic design condition, bleeding is a critical concern that must be addressed. Song et al. achieved a maximum surface texture depth of 4 mm for FMA-13 through rational filling degree design [29]. This indicates that the asphalt mastic liquid level should be maintained below the skeleton structure during design, so that under compaction loads, the load is primarily carried by the skeleton. This approach prevents asphalt bleeding during service and ensures skid-resistant durability. Xu et al. applied the MaFF method to design FMA-20 and used it in a crack repair project, verifying the constructability of this mixture [30]. Collectively, the above findings confirm that the FMA mixture is both scientifically sound and practically feasible, spanning from material design to engineering application.

The current skeleton design of FMA mixtures predominantly relies on discontinuous gradation, with mix design calculations based largely on the dry tamping method and volumetric analysis. This approach is built upon a fundamental assumption that fine aggregates serve only as fillers and do not interfere with the coarse aggregate skeleton. In practice, however, even fine aggregates of small particle size can exert an influence on the formation of the skeleton structure during compaction. Consequently, the theoretical foundation of existing methods remains insufficiently rigorous, and their engineering application depends heavily on empirical experience. Moreover, existing measurements of the void in the aggregate are primarily directed at coarse aggregates, while test methodologies that account for the presence of fine aggregates remain unavailable. This is mainly due to the fact that fine aggregates are prone to segregation without asphalt binder, making accurate measurement of the voids in the aggregate unattainable.

In view of the above problems, this study proposes a deviation coefficient method based on the maximum density curve of n=0.45 in the Talbot formula [31]. This method realizes the flexible design of the discontinuous gradation skeleton by reducing the passing rate at the 4.75 mm key sieve, providing a quantitative basis for tuning the aggregate packing density. In order to accurately evaluate the stacking state of the skeleton designed by the deviation coefficient method, a wet mixing test was further proposed to determine the voids in the aggregate ($V_{ag}$) of different skeletons. It addresses the segregation and lubrication limitations of the conventional dry tamping approach. Subsequently, this method was used to systematically determine the 11 gradations designed by the deviation coefficient method, and a quantitative relationship model between the deviation coefficient and $V_{ag}$ was established. In addition, in order to clarify the difference between the $V_{ag}$ of the wet mixing test and the $V_{ag}$ of the FMA mixture, this study introduced the CT scanning three-dimensional reconstruction method for comparative analysis, calibrated the wet mixing test results, and determined the reasonable value range of the reduction coefficient [32]. Finally, based on the research findings, the MaFF method is systematically refined to form a complete skeleton design framework. This study contributes to the field by providing a theoretically grounded and experimentally validated approach for the quantitative design and evaluation of FMA aggregate skeletons.

## 2. Raw Materials

### 2.1. Asphalt

High-viscosity asphalt was chosen as the binder in this study, consistent with the material selection in existing FMA-13 studies. The test results of the high-viscosity modified asphalt are shown in Table 1, and all technical indicators met the requirements of the *Technical Specifications for the Construction of Highway Asphalt Pavements* (JTG F40-2004).

### 2.2. Aggregate and Filler

The aggregate used in this study is diabase, and the filler is limestone power. The test results of aggregate and filler are shown in Table 2, and all the technical indicators met the requirements of JTG F40-2004.

## 3. Mix Design and Test Method

### 3.1. Mix Design

Fluid mastic asphalt (FMA) fills the voids in the aggregate by fluid asphalt mastic, so that the internal air voids are less than 1%. At the same time, relying on the reasonable skeleton design to resist the vehicle load, the high temperature stability of the mixture is ensured [22]. At present, the performance advantages of FMA have been verified by many studies. The Mortar Flow-Filling Method (MaFF) is mainly used in the design. The core idea of this method is to leverage the flow characteristics of the asphalt mastic during the molding process, thereby breaking away from the long-standing design concept of traditional hot mix asphalt (HMA), which requires the mastic to be viscous (no leakage).

The MaFF method consists of two parts: skeleton design and mastic design. Its basic principle is that during the molding process, the skeleton forms a stable load-bearing system by interlocking between particles, primarily to withstand vehicle loads. Additionally, the mastic in a flowing state, leveraging its own fluidity and external forces, fully fills the voids in the aggregate during compaction, thereby achieving skeleton-ultra-dense structure. The fluidity of the mastic is influenced by two main factors. The first is the flow characteristics of the asphalt mortar, which are primarily determined by the temperature and the properties of the asphalt. The second is the content of asphalt mortar in the mixture. The higher the content of free-flowing mortar in the mixture, the better is the fluidity of the mortar during the molding process. Therefore, the skeleton design not only affects the stability of the skeleton structure but also enables the adjustment of the asphalt content over a wide range according to functional requirements by controlling the voids in the aggregate. This highlights that the skeleton design is crucial to the performance of FMA mixtures.

Traditional HMA mixtures are primarily designed based on the maximum density curve. This curve was first proposed by W.B. Fuller et al. and later improved by Talbot, resulting in the Talbot formula, as shown in Equation (1) [33].

(1) $$P_{i}=100\times {\left(\frac{d_{i}}{D}\right)}^{n},$$

where $P_{i}$ is the passing rate (%) of $d_{i}$, D is the maximum particle size (mm), and n is the curve index. When 0.45 is taken, the density of the asphalt mixture is usually the largest.

**Figure 1:**
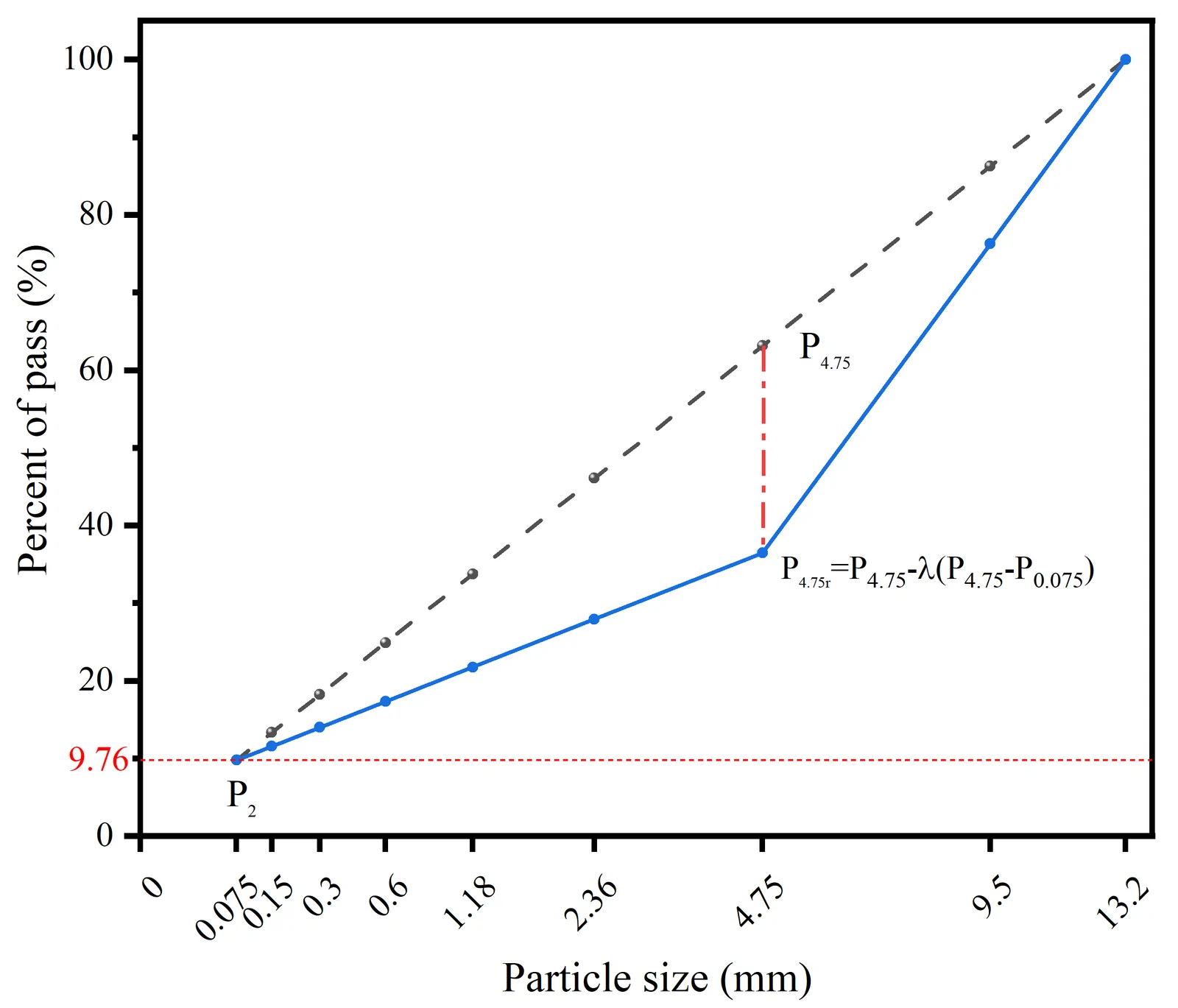
Relationship between gradation curve and deviation coefficient.

To adjust the gradation curve shape by reducing the passing percentage of key sieve sizes (e.g., 4.75 mm or 2.36 mm), thereby controlling the voids in the aggregate, this paper proposes the deviation coefficient method. In the gradation curve with 0.45 power of the sieve size, the maximum density curve is a straight line, as shown in Figure 1. The deviation coefficient method takes the maximum density curve with n=0.45 as the reference and uses λ to represent the degree to which the passing percentage at the key sieve deviates from the maximum density line. The deviation coefficient ranges from 0 to 1. The smaller the deviation coefficient, the closer the gradation is to the maximum density line. By changing the deviation coefficient, the discontinuous gradation mixture with different voids in the aggregate can be designed to adapt to the design of different asphalt mastic contents, so as to flexibly adjust the asphalt content of the FMA based on the needs of the functional pavement.

The specific procedure of the deviation coefficient method is as follows. According to the Talbot formula, when n = 0.45, the passing percentages of each aggregate size can be calculated as shown in Table 3.

After converting the abscissa to $d_{i}^{0.45}$, the gradation curve becomes a straight line. For asphalt mixtures with a nominal maximum aggregate size of 13.2 mm, the 4.75 mm sieve serves as the critical dividing point between coarse and fine aggregates. Therefore, by adjusting the passing rate at this sieve, the proportion of coarse to fine aggregates can be effectively regulated, thereby significantly influencing the interlocking characteristics of the skeleton structure. Taking 4.75 mm as the key sieve as an example, the passing rate of the reduced passing rate is calculated according to Equation (2).

(2) $$P_{4.75r}=P_{4.75}-\lambda \left(P_{4.75}-P_{0.075}\right),$$

where λ is the deviation coefficient. The value range is from 0 to 1, and the spacing is 0.1. $P_{4.75r}$ is the passing rate after the conversion of the key sieve (%). $P_{0.075}$ is the passing rate of the unconverted 0.075 mm (%). $P_{4.75}$ is the pass rate of the unconverted 4.75 mm (%).

For the passing rate of the rest of the aggregate, the linear interpolation method is used for the determination. The linear interpolation is performed according to $P_{13.2}$, $P_{4.75r}$ and $P_{0.075}$, and the converted gradation curve is obtained, as shown in Figure 1.

**Figure 2:**
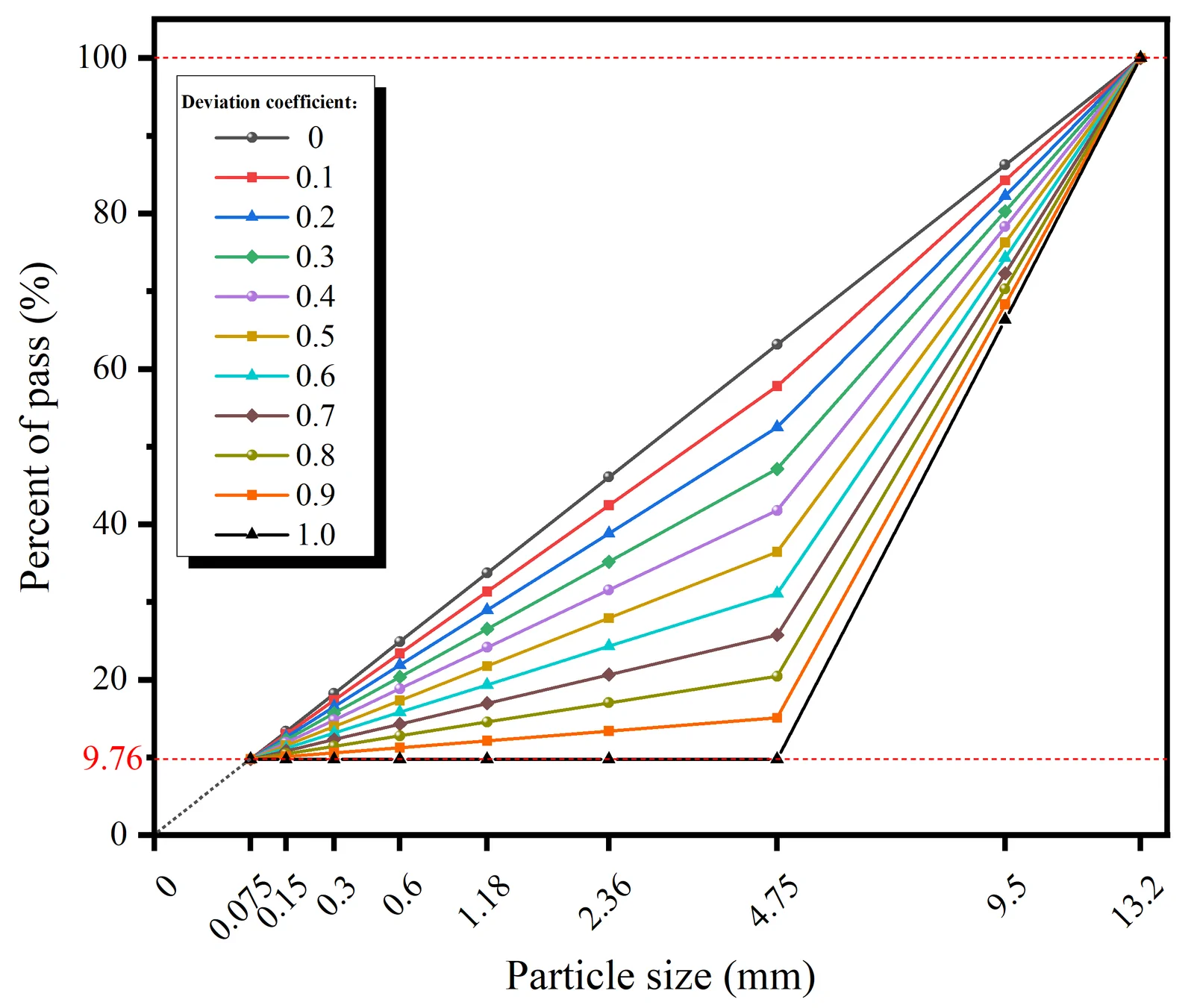
Passing percentage of aggregate fractions by deviation coefficient method (horizontal coordinate $d^{0.45}$).

According to the above method, when the λ gradually increases from 0 to 1.0 (0.1 step size), the pass rate of each aggregate is shown in Table 4 and Figure 2.

It can be seen from Table 4 and Figure 2 that as λ gradually increases from 0 to 1.0, the passing rate of 4.75 mm continues to decrease, resulting in a regular change in the grading curve. The passing rate of aggregates with different particle sizes decreases with the increase in λ, but the decrease range is obviously different. Among them, the pass rate of the coarse aggregate part, such as 9.5 mm, decreased slightly, while the pass rate of the fine aggregate part of 2.36 mm and below decreased significantly. This is because the deviation coefficient directly controls the pass rate of 4.75 mm. The smaller the λ is, the higher the pass rate of 4.75 mm and the greater the fine aggregate content.

### 3.2. Test Methods

The above gradation design is mainly based on the deviation coefficient method for theoretical calculation. After determining the gradation, the key step is to determine the voids in the aggregate ($V_{ag}$). In view of the coarse aggregate void, Wu et al. used the dry tamping method for measurement [34]. In the coarse aggregate, the aggregates support each other to form a skeleton structure during the dry tamping process, and there is no obvious segregation phenomenon. However, when the fine aggregate is added to form the skeleton structure, the fine aggregate will gradually sink to the bottom during the dry tamping process, resulting in serious segregation, which is completely inconsistent with the skeleton structure in the asphalt mixture. In addition, during the dry tamping process, the aggregate particles are in a dry friction contact state. Due to the rough surface of the coarse aggregate and the large friction resistance, it is difficult for the particles to reach the ideal dense state. In order to solve the above two problems, this paper proposes the use of the wet mixing test to determine the $V_{ag}$.

However, if too little asphalt is added in the wet mixing test, the bonding ability to fine aggregate is insufficient, and the lubrication effect is limited. If too much asphalt is added, excess free asphalt may be produced, which interferes with the interlocking arrangement between aggregates and affects the accuracy of the measurement results. Therefore, it is very important to reasonably select the amount of asphalt in the wet mixing test. The rationality for evaluating the amount of asphalt mainly considers three aspects: the degree of adhesion between the asphalt and the aggregate, the dense state of the skeleton structure, and the simplicity of the test operation.

Based on the above considerations, this study designed four sets of control tests with different asphalt contents, where the asphalt–aggregate ratios were set to 1%, 2%, 3% and 4%, respectively. The test used the same grade of aggregate, and each group of asphalt–aggregate ratio formed three parallel specimens in which the weight of the specimens remained the same. After the specimen was cooled and demolded, the height of the specimen was measured in four directions with a vernier caliper, and the average value was taken as its height. Then, the volume of the specimen was calculated according to the volume formula, and the $\gamma_{c}$ of the specimen was obtained. Finally, the $V_{ag}$ of each specimen was calculated by Equations (3) and (4).

(3) $$V_{ag}=\left(1-\frac{\gamma_{c}}{\rho_{c}}\right)\times 100\%,$$

(4) $$\gamma_{c}=\frac{m_{s}-\;m_{a}}{V_{s}-\;V_{a}},$$

where $V_{ag}$ is the voids in the aggregate (%). $\gamma_{c}$ is the compact packing density of the specimen (g/cm^3^). $\rho_{c}$ is the synthetic bulk density of the aggregate (g/cm^3^). $m_{s}$ is the mass of the specimens (g). $m_{a}$ is the mass of the asphalt (g). $V_{a}$ is the volume of the asphalt (cm^3^). $V_{s}$ is the volume of the specimens (cm^3^).

In addition to examining the amount of asphalt added, this paper also analyzes the number of tamping times and the number of compaction times in the wet mixing test. For the 1 tamping in the specification, the process is as follows: tamping 15 times along the periphery of the mold with a ramming rod and then tamping 10 times in the middle area. In this study, the different tamping times after loading were systematically compared, and the three cases of 0 tamping, 1 tamping and 2 tamping were investigated. In addition, the number of blows directly affects the compactness of the specimen. In this study, three compaction times of 50 times, 75 times and 125 times were tried. Three specimens were formed for each time, and the remaining conditions remained unchanged. The $V_{ag}$ was measured, and the average value was taken.

The molding process of this study adopts the Marshall compaction method [28]. The test steps are as follows.

**Figure 3:**
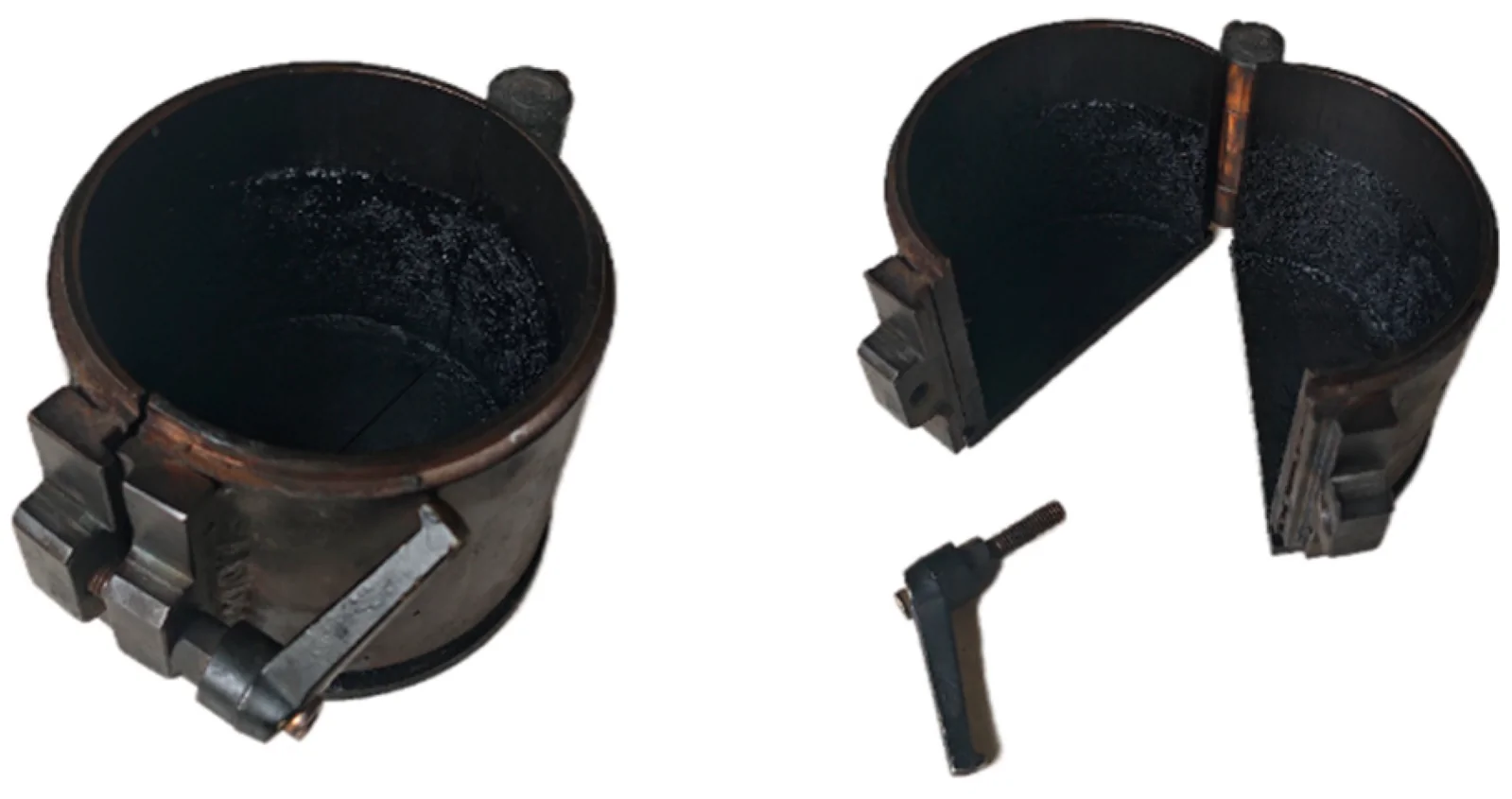
Single-face compaction Marshall mold.

(1) According to the viscosity temperature curve obtained from the asphalt rotational viscosity test, the temperature of 200 °C corresponding to an asphalt viscosity of 0.28 Pa·s is used as the test mixing temperature.
(2) Put the asphalt, aggregate, and molds into the oven and keep them at 200 °C for 4 h. The 4 h conditioning period is intended to ensure sufficient preheating to achieve thermal equilibrium at the target temperature while avoiding asphalt aging due to excessive heating time.
(3) Pour the heated aggregate into the mixer and then mix for 90 s. Add a% asphalt and continue to mix for 90 s until the mixture is uniform. The mixing temperature should be kept at 200 °C.
(4) The aggregate is filled in two times. According to the Marshall test, the weight of the standard Marshall specimen is usually about 1200 g. The mold of the test is shown in Figure 3. However, the pre-test shows that when the n is large, the coarse aggregate content of the corresponding gradation increases significantly. If the mixture of 1200 g is still used, aggregate overflow will occur during the molding process. In order to ensure the stability of the molding operation and the consistency of the specimen size, the weight of the specimen was finally determined to be 1100 g. The total weight of each specimen is controlled at 1100 ± 5 g.
(5) Place the loaded mold in the Marshall compaction apparatus and compact it c times. The compacted specimen should be naturally cooled in a cool place for 24 h before demolding.
(6) Measure the height of the specimen from four directions using a vernier caliper and take the average as the height. If the difference between the maximum and minimum heights measured is greater than 1 mm, the specimen needs to be remolded.
(7) Calculate the volume of the specimen based on the measured height and then determine the $\gamma_{c}$. Finally, calculate the $V_{ag}$ according to Equations (3) and (4).

## 4. Discussion and Analysis

### 4.1. Determine the Parameters of the Wet Mixing Test

**Figure 4:**
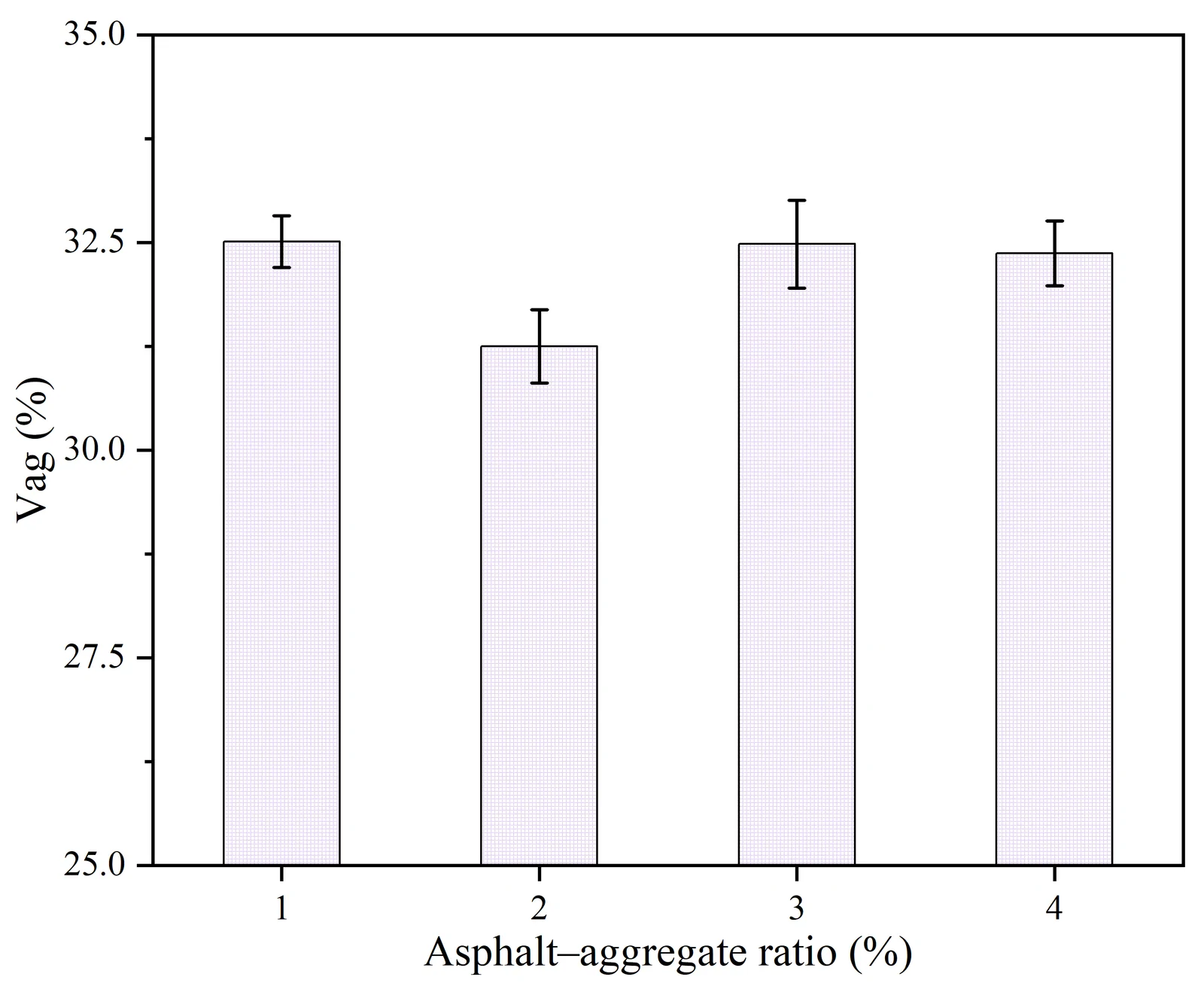
$V_{ag}$ of specimens under different asphalt–aggregate ratios.

**Figure 5:**
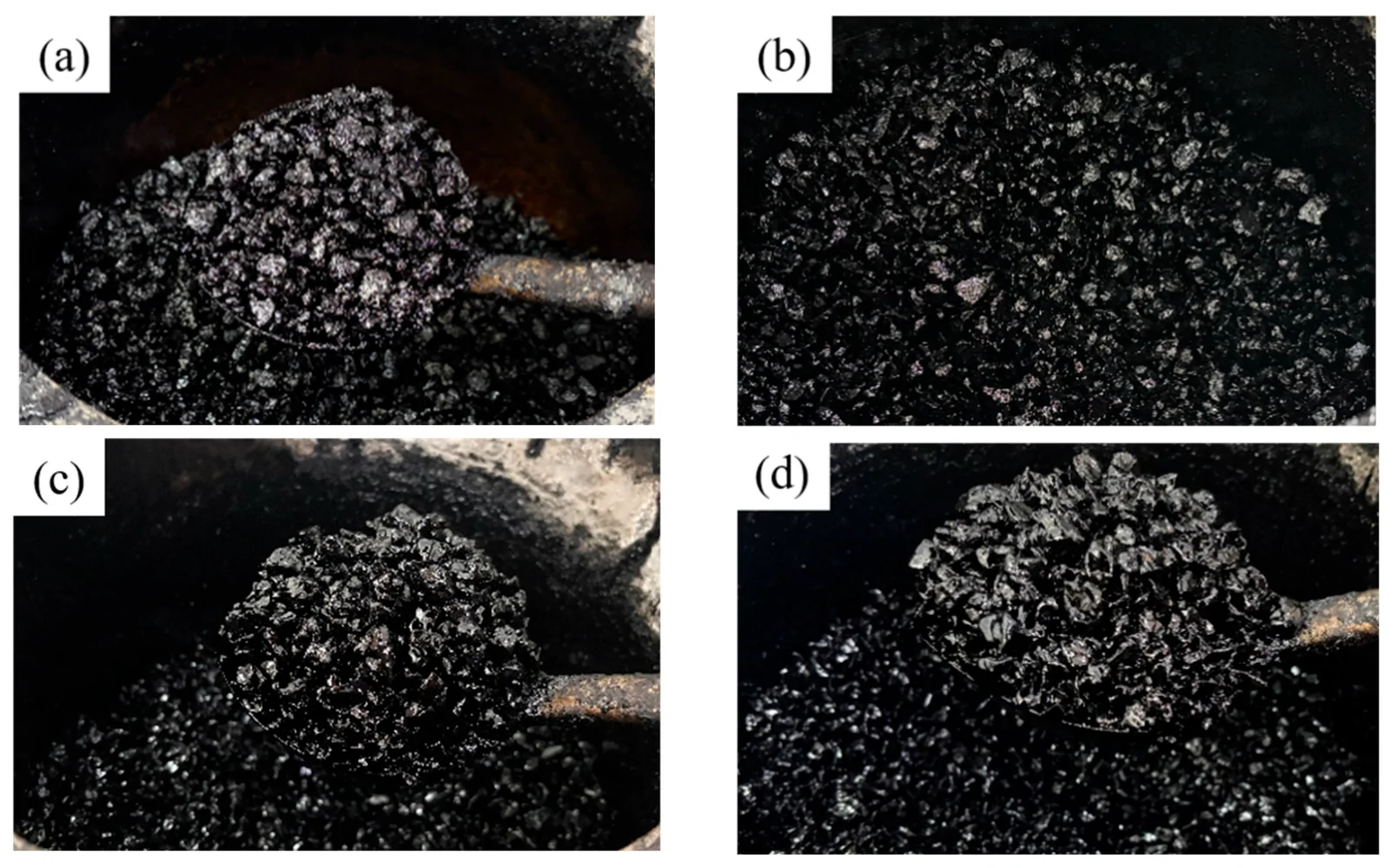
The state under different asphalt–aggregate ratio conditions: (**a**) 1%, (**b**) 2%, (**c**) 3%, (**d**) 4%.

In order to determine the parameters of the wet mixing test, the asphalt–aggregate ratio, the number of tamping strokes and the number of blows were analyzed. Figure 4 shows the influence of different asphalt–aggregate ratios on $V_{ag}$, and Figure 5 shows the coating of asphalt on the surface of the aggregate under different asphalt–aggregate ratios.

It can be seen from Figure 4 that when the asphalt–aggregate ratio is 2%, the test results are the most ideal. Compared with the specimens formed under the other three asphalt–aggregate ratios, the $V_{ag}$ of the 2% sample is the smallest, indicating that the specimen is most dense. At the same time, it can be seen from Figure 5 that when the asphalt–aggregate ratio is 1%, the surface of some aggregates is not coated with asphalt, indicating that it cannot reach the best state. When the asphalt–aggregate ratio is 3% or 4%, although the aggregate surface has been completely coated with asphalt, there is more free asphalt in addition to the asphalt adhered to the aggregate surface. During the forming process, the free asphalt may cause the volume of the specimen to expand, making the measured volume larger, so that the calculated $V_{ag}$ is also larger. Therefore, the asphalt–aggregate ratio of 2% was finally determined as the optimal asphalt content of the wet mixing test.

**Figure 6:**
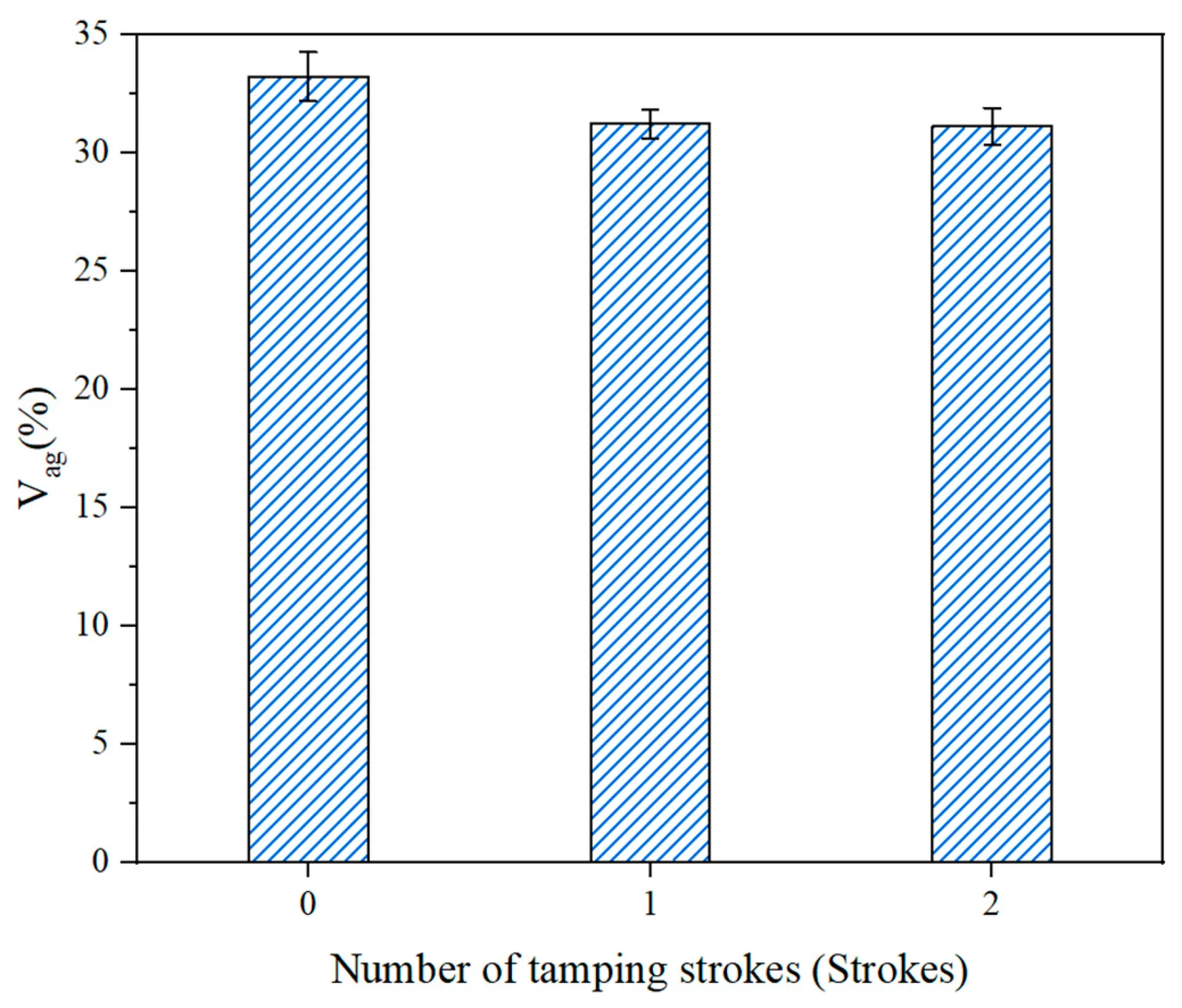
$V_{ag}$ of specimens under different tamping strokes.

Figure 6 shows the $V_{ag}$ under different tamping strokes.

From Figure 6, it can be seen that the $V_{ag}$ of the untamped specimen is significantly higher than that of the rammed specimen, indicating that the tamping operation helps the aggregate to be evenly arranged and provides a more ideal compaction state for subsequent compaction. The difference between the $V_{ag}$ obtained by tamping one and two times is very small. From a numerical point of view, tamping one time seems to be sufficient. However, it is found that if the 1100 g mixture is poured into the mold at once and tamped for practice, it is easy to cause aggregate overflow. The problem can be effectively avoided by using the method of loading twice. The specific operation is as follows. Firstly, about 550 g of mixture is loaded, and the preliminary tamping is carried out. Then, the remaining part is loaded, and the final tamping is completed, so as to ensure that the aggregate does not overflow and the operation is stable and controllable. Therefore, it is finally determined to use two loading times with tamping one time after each loading based on the operation convenience and test stability. The total number of tamping times is 2 times.

**Figure 7:**
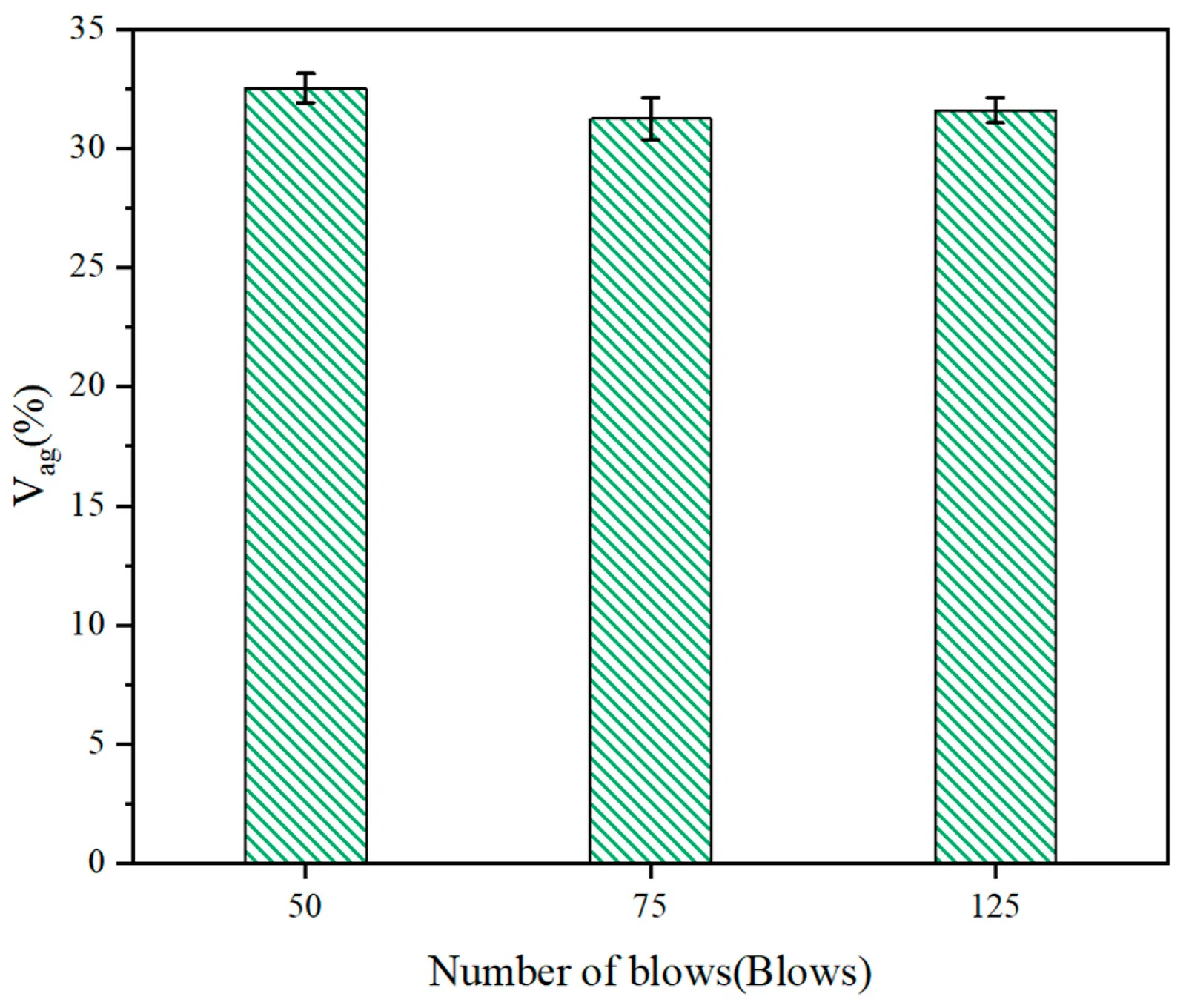
$V_{ag}$ of specimens under different blows.

Figure 7 shows the $V_{ag}$ of the specimen under different blows.

It can be seen from Figure 7 that the $V_{ag}$ is the largest when the number of blows is 50, indicating that the specimen has not yet reached the dense state. The $V_{ag}$ values of blows of 75 times and 125 times are very close, indicating that 75 times can cause the aggregate skeleton to reach a stable state. The effect of increasing the number of blows on the compactness is limited. From the perspective of operational efficiency, the number of blows in the Marshall compaction method was finally determined to be 75 times.

Figure 5 summarizes the test results of the wet mixing test and its corresponding coefficient of variation. The data show that even though the maximum coefficient of variation is only 2.39%, indicating that the dispersion of the test data is low, the test results have good repeatability and consistency. This provides strong data support for the rationality and reliability of the wet mixing test as a skeleton accumulation state evaluation method.

### 4.2. Analysis of the Test Results of the V_ag_

Based on the in-depth analysis of the wet mixing test in Section 4.1, all the test parameters were finally determined. In this section, according to the design of Section 3.1, the wet mixing test parameters determined in Section 4.1 and the steps in Section 3.2 are strictly used to form the specimens. The height of the different skeleton structures is measured, and the $V_{ag}$ is calculated. The results and their standard deviations are shown in Table 5.

**Figure 8:**
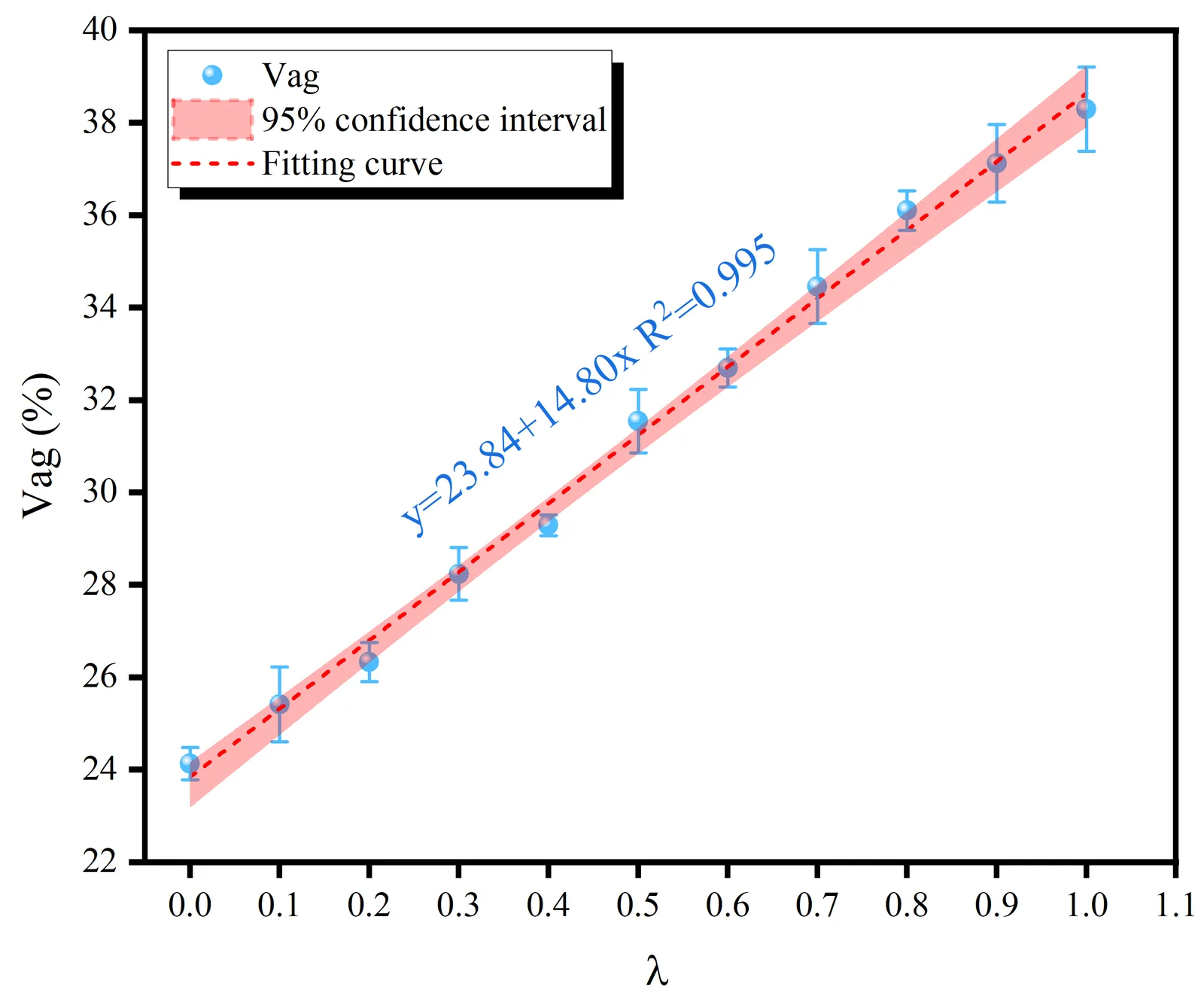
Deviation coefficient method.

It can be seen from Table 6 that as the λ gradually increases from 0 to 1.0, the $V_{ag}$ continues to rise from 24.13% to 38.29%. When λ=0, the $V_{ag}$ is the Talbot curve when n=0.45. At this time, the $V_{ag}$ is the smallest (24.13%), the fine aggregate content is the highest, and the gradation is the finest. When λ=1, the pass rate of 4.75 mm decreased to 9.76%, the coarse aggregate content increased significantly, and the $V_{ag}$ reached the maximum value of 38.29%. There is a good linear relationship between $V_{ag}$ and λ, as shown in Figure 8.

By linear regression fitting of $V_{ag}$ and λ, the fitting equation is obtained:

(5) $$V_{ag}=14.80\lambda +23.84,\;R^{2}=0.996$$

The fitting results show that there is a good linear correlation between λ and the $V_{ag}$ (R^2^ > 0.99). The linear relationship intuitively reflects the control law of the deviation coefficient method. The smaller λ is, the smaller the $V_{ag}$. The larger λ is, the larger the $V_{ag}$ is, and the higher the asphalt–aggregate ratio corresponding to the FMA can be designed.

Figure 8 shows the regression curve and its 95% confidence interval based on the experimental data fitting. In order to further evaluate the predictive ability of the model, we extracted the relevant data and drew a comparison diagram between the predicted interval shown in Table 7 and the measured value of the test. It can be seen from the figure that in the 95% prediction interval, the measured values of eight $V_{ag}$ all fall within the prediction range. Although some points deviate slightly, the deviation range is very small. This indicates that the fitting curve can well reflect the relationship between λ and $V_{ag}$.

The gradation obtained by the deviation coefficient method shows a clear linear relationship between $V_{ag}$ and the design parameters, which is convenient for rapid estimation and preliminary design. However, it should be noted that the deviation coefficient method is based on the maximum density curve of n=0.45, and the generated gradation form basically changes near the reference curve. The control range is relatively limited, and it is more suitable for fine-tuning near the reference gradation.

It should be emphasized that the confidence and prediction intervals reported herein are derived from the same dataset used to establish the empirical relationship between λ and $V_{ag}$. Consequently, while these intervals provide useful information about the descriptive accuracy and uncertainty of the fitted relationship within the tested gradation range, they do not constitute independent validation of the model’s predictive capability. Independent validation would require additional experimental data from a separate material system or gradation range, which is a key direction for future work.

### 4.3. Comparative Analysis of the $V_{ag}$ Between Wet Mixing Test and CT Scanning Three-Dimensional Reconstruction Method

The $V_{ag}$ was analyzed according to the wet mixing test. However, there will be some differences between the $V_{ag}$ measured by the wet mixing test and the FMA mixtures. Therefore, in order to obtain the differences between them, this paper analyzes the $V_{ag}$ of the FMA mixtures by the CT scanning three-dimensional reconstruction method [35].

The CT scanning three-dimensional reconstruction method provides a new technical means for the accurate determination of the $V_{ag}$. The basic principle is to use X-rays to scan the specimen layer by layer to obtain continuous high-resolution two-dimensional slice images. Then, these images are reconstructed into three-dimensional digital models by three-dimensional reconstruction software, such as Avizo3D, so as to realize the visualization and quantitative analysis of the internal structure of the specimen [36].

**Figure 9:**
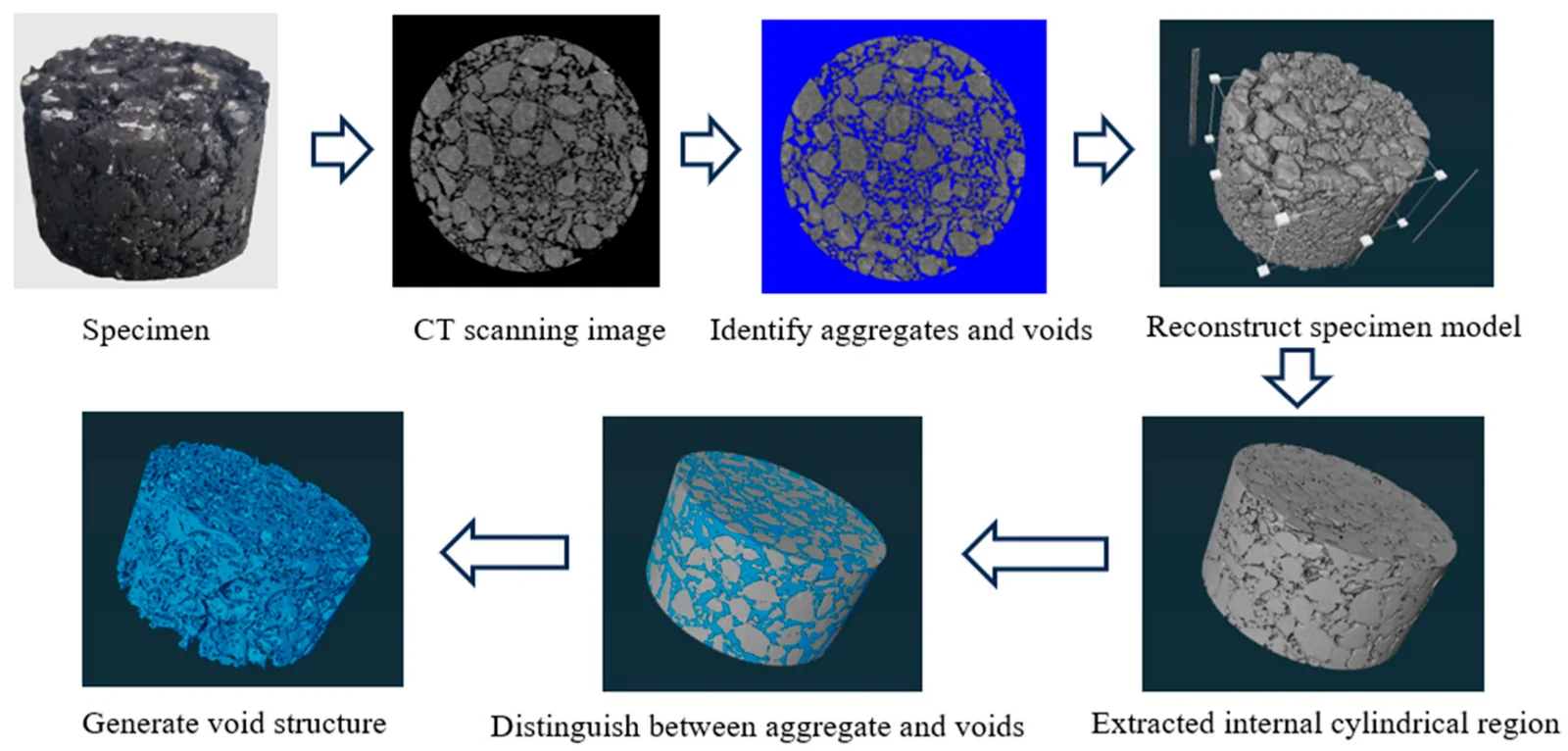
CT scanning three-dimensional reconstruction method.

In the specific operation, the specimens were first placed in the CT scanning system and continuously scanned along the height direction to obtain thousands of slice images. The CT scanning was performed using a Bruker SkyScan 2211 high-resolution X-ray micro-CT (Bruker, Bilerica, MA, USA). The CT scanning was performed at a voltage of 220 kV and a current of 200 μA, with a resolution of 53 μm. Sequential two-dimensional cross-sectional images were acquired at an inter-slice spacing of 53 μm. Regarding threshold selection, a grayscale threshold range of 0–45 was determined through systematic testing, which effectively captures the structural characteristics of the aggregate skeleton. After importing the images into Avizo3D, the digital model is generated by the three-dimensional reconstruction algorithm. In order to eliminate the influence of the boundary effect, the internal cylinder area can be extracted from the model as a sub-model to represent the real accumulation state inside the specimen. The steps are shown in Figure 9.

**Figure 10:**
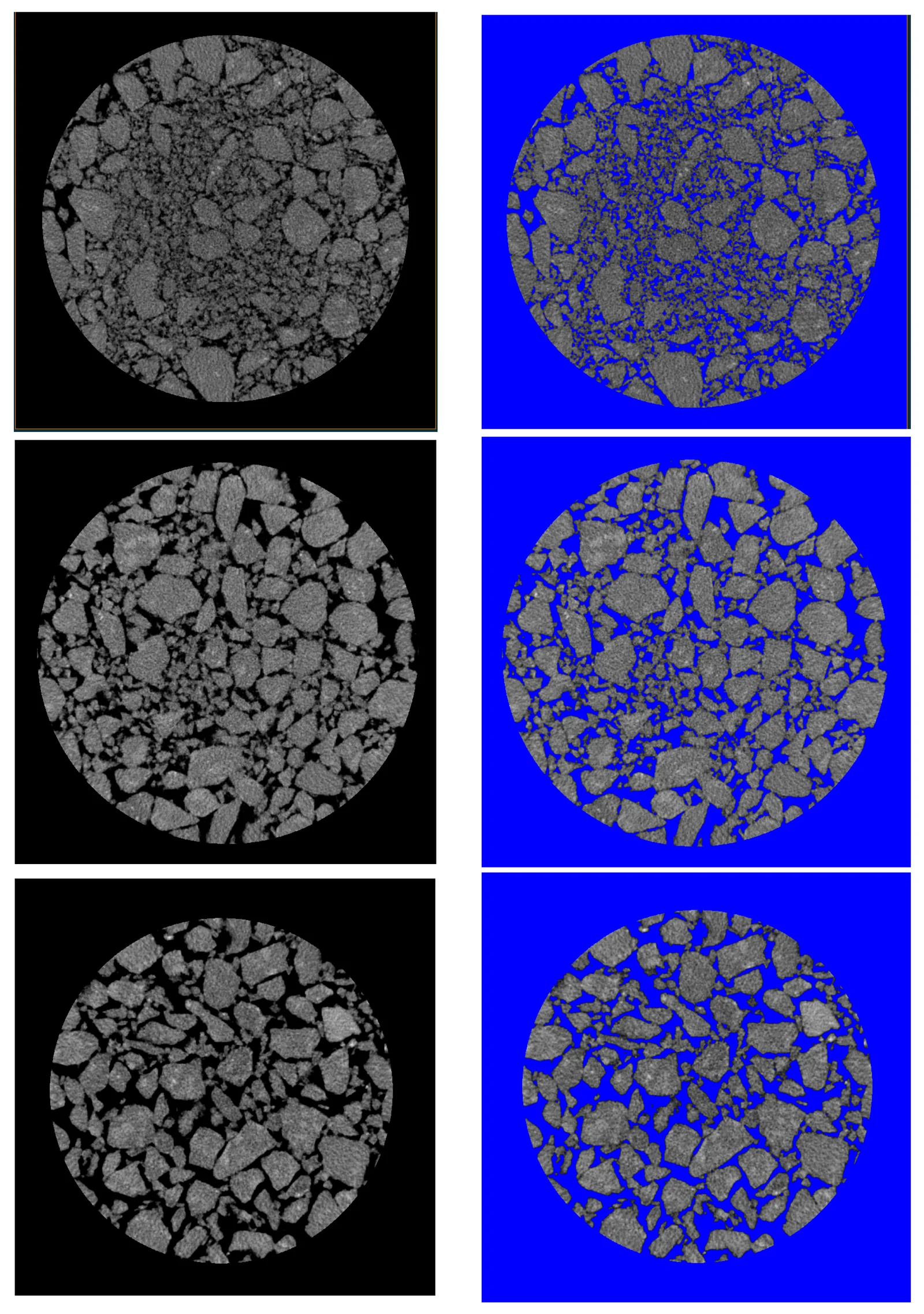
The $V_{ag}$ between the original two-dimensional images and the images generated by Avizo3D. (λ is 0.1, 0.5 and 0.7, respectively.).

As shown in Figure 10 and Table 8, the slice images of three layers were selected for comparative observation. It can be seen that the Avizo3D 2019 software can clearly and accurately identify the void area in the image. The sum of the volume of the air voids and the asphalt mastic is the volume of the voids in the aggregate. The gray difference between the mastic and the aggregate and air voids is obvious, and the recognition results by Avizo3D are basically consistent with the visual judgment. The results show that the CT scanning three-dimensional reconstruction method has good accuracy in air voids, which verifies the reliability of this method in the quantitative analysis of $V_{ag}$.

**Figure 11:**
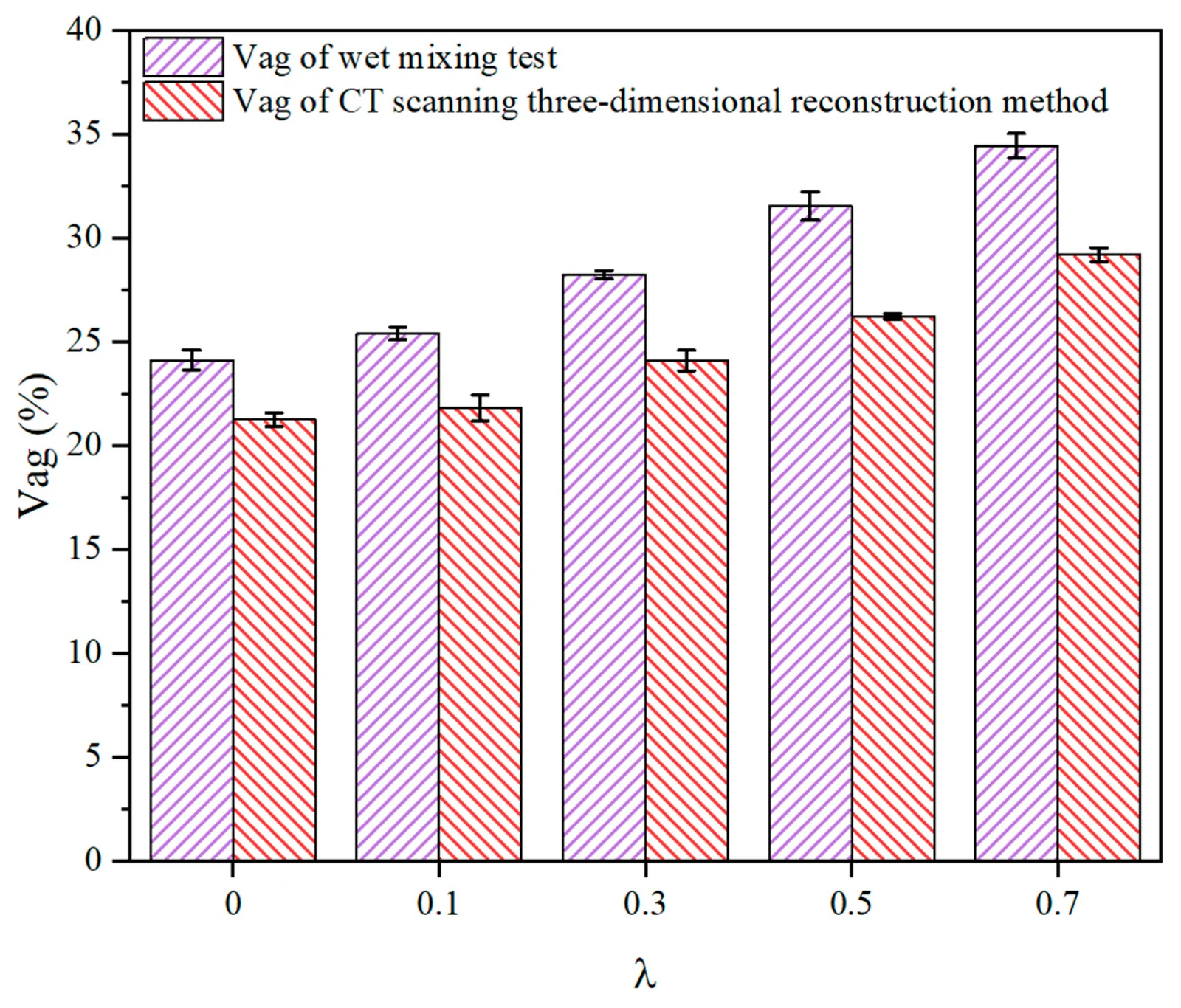
$V_{ag}$ of wet mixing test and of CT scanning three-dimensional reconstruction method.

The software counts the number of pixels in the marked air voids and calculates the $V_{ag}$ in combination with the total volume of the model. The specific results are shown in Figure 11.

Figure 11 compares the $V_{ag}$ measured by the wet mixing test and the CT scanning three-dimensional reconstruction method under different deviation coefficients. As λ increases from 0 to 0.7, the $V_{ag}$ measured by both methods shows an upward trend. However, the $V_{ag}$ measured by the CT scanning three-dimensional reconstruction method is lower than that of the wet mixing test under each λ. The ratio of the two is stable between 0.83 and 0.86, with an average of 0.84. This is mainly because the object of the wet mixing test is the aggregate skeleton. Its voids are completely open and not filled. The CT scanning three-dimensional reconstruction method is aimed at the asphalt mixture. The voids are filled with fluid mastic, which has a certain lubrication effect in the molding process. It is conducive to the rearrangement of the skeleton particles, so that the $V_{ag}$ is lower. Therefore, the $V_{ag}$ measured by the wet mixing test should not be directly equivalent to the $V_{ag}$ in the FMA mixture but should be multiplied by a reduction coefficient for conversion. Based on the ratio results of each group of data, 0.8–0.9 is taken as the conversion coefficient between the results of the wet mixing test and the results of the CT scanning three-dimensional reconstruction method. The coefficient can be used as an empirical correction parameter for the rapid evaluation of the skeleton state of the FMA mixture by the wet mixing test, which provides a reference for the application of the wet test results to the MaFF method.

### 4.4. Design of MaFF Method Based on Deviation Coefficient Method

**Figure 12:**
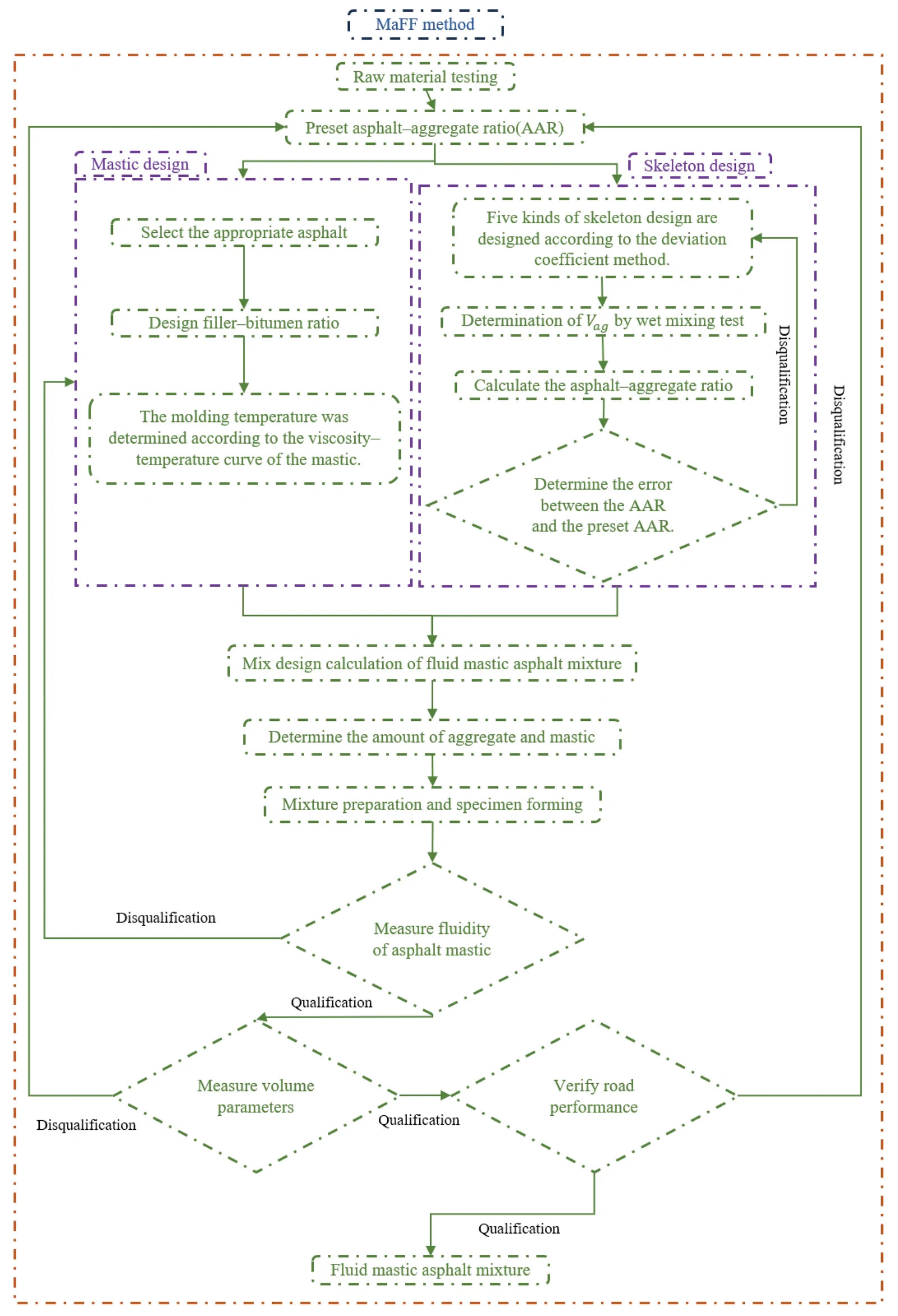
Design flow chart of MaFF method based on deviation coefficient method and wet mixing test.

Zheng et al. reported that the skeleton design of the MaFF method mainly relies on the dry tamping method to determine the voids in the coarse aggregate. The fine aggregate part is based on the concept of discontinuous gradation, assuming that it only plays a filling role and does not interfere with the coarse aggregate skeleton. Under this framework, the initial design of the skeleton structure mainly depends on theoretical calculation, and the complexity of the fine aggregate behavior in the compaction process is not fully considered. However, Cui et al. showed that fine aggregates are not simply filled, and they will be rearranged due to lubrication and compaction effects in the presence of asphalt mastic, thereby changing the contact state between coarse aggregates and the $V_{ag}$ [34]. Therefore, it is difficult to accurately predict the skeleton performance of the asphalt mixture only by the dry tamping method and theoretical calculation. It is necessary to introduce the wet mixing test to repeatedly check and adjust the skeleton parameters. The design flow chart is shown in Figure 12.

As shown in Figure 12, the verification process of this skeleton design is as follows. Firstly, according to the preset design asphalt–aggregate ratio, five different skeleton structures are designed by using the deviation coefficient method. Secondly, the $V_{ag}$ of each skeleton was measured by the wet mixing test, and the corresponding asphalt–aggregate ratio was calculated based on the calculation formula of the MaFF method. Subsequently, the asphalt–aggregate ratio is compared with the preset oil–stone ratio, and the relative error between the two is calculated. When the error is controlled within 0.1%, it is considered that the skeleton design meets the target requirements.

This design process not only puts forward a clear theoretical basis and test methods for each stage but also ensures the feasibility of the design objectives through closed-loop verification. The process combines theoretical calculation with experimental correction, which has strict logic and clear hierarchy.

Taking the FMA-13 asphalt mixture (with a nominal maximum aggregate size of 13.2 mm, a design asphalt–aggregate ratio of 7%, and a filler–asphalt ratio of 1.0) as an example, the mix design procedure of the proposed method is described in detail below.

According to the definition of the asphalt–aggregate ratio, the design asphalt–aggregate ratio is directly determined as 7%. Based on Equations (6) and (7) in the MAFF method, the mix composition can be further calculated:

(6) $$P_{ag}+P_{ma}=100$$

(7) $$\frac{P_{ag}\times \left(V_{ag}-VV\right)}{100\times \;\gamma_{c}}\times F_{d}=\frac{P_{ma}-(1-\;F_{d})P_{ma0}}{\gamma_{ma}}$$

where $P_{ag}$ is the aggregate content of the FMA mixture (%); $P_{ma}$ is the asphalt content (%); $P_{ma0}$ is the amount of adhesive asphalt mastic (%); VV is the design air voids (%); $\gamma_{ma}$ is the synthetic relative density of the asphalt mastic; $\gamma_{c}$ is the bulk density of the aggregate in the tamping or compaction state; $\gamma_{ag}$ is the effective relative density of the synthetic aggregate, which can be replaced by the relative density of the synthetic bulk volume in rough calculation; and $F_{d}$ is the filling degree, which indicates the filling degree of the asphalt mastic to the voids in the aggregate. The $V_{ag}$ is the percent voids in the skeleton aggregate in the FMA mixture (%). The $F_{d}$ is 1.0 in this study.

Considering that it is difficult to achieve absolute zero porosity within the asphalt mixture, the design air voids are recommended as VV=0.5%. The relative density of the asphalt used in this study is 1.03, and the apparent relative density of the mineral filler is 2.894. With a filler–asphalt ratio of 1.0, $\gamma_{ma}$ is calculated to be 1.519. Based on Equations (6) and (8), $P_{ma}=13.08\%$ and $P_{ag}=86.92\%$ are obtained:

(8) $$P_{ma}=\frac{AAR}{1+AAR}\times \left(1+FAR\right)\times 100\%$$

where AAR is the asphalt–aggregate ratio (%), and FAR is the filler–asphalt ratio.

Once the λ value is determined, the gradation composition of the mixture can be obtained from Table 4, while the $V_{ag}$ of the skeleton structure can be determined from Figure 8. During the design process, iterative approximation is performed by selecting corresponding values from these two figures. The detailed iteration procedure is omitted here, and only the final mix composition is presented.

According to Table 7, when λ=0.2, the $V_{ag}$ measured by the wet mixing test is 26.33%. Considering the systematic deviation between the 3D reconstruction results and the wet mixing test, a reduction coefficient of 0.84 (the average value obtained in this study) is applied. Thus, the $V_{ag}$ substituted into Equation (7) is: $V_{ag}=26.33\times 0.84=22.12$.

Based on the gradation passing rates in Table 4 and the density data in Table 9, the bulk specific gravity of the synthetic aggregate is calculated as 2.855, and $\gamma_{c}$ = 2.233, using Equations (9)–(11).

(9) $$\gamma_{b}=\frac{100}{\frac{P_{1}}{\gamma_{1}}+\frac{P_{2}}{\gamma_{2}}+\ldots +\frac{P_{n}}{\gamma_{n}}}$$

(10) $$\rho_{c}=\gamma_{b}\times \rho_{T}$$

(11) $$V_{ag}=\left(1-\frac{\gamma_{c}}{\rho_{c}}\right)\times 100$$

where $\gamma_{b}$ is the bulk specific gravity of the combined aggregate mixture; $P_{1}$, $P_{2}$, …, $P_{n}$ are the percentages of each aggregate fraction in the total mass of the combined aggregate mixture, summing to 100%; $\gamma_{1}$, $\gamma_{2}$, …, $\gamma_{n}$ are the bulk specific gravities of each aggregate fraction; $\rho_{T}$ is the density of water at the test temperature T (g/cm^3^), with $\rho_{T}$ = 0.99702 at 25 °C; and $\rho_{c}$ is the synthetic bulk volume relative density.

Substituting the above parameters into Equation (7) for verification, the left and right sides of the equation are equal, confirming the validity of the mix design. Subsequent validation through pavement performance tests is required. This method significantly reduces trial-and-error costs and improves both the efficiency and standardization of the mix design process.

## 5. Conclusions

This study focuses on the design and evaluation of FMA and conducts systematic research from five aspects: gradation regulation, test method, quantitative model, correction and design process. Firstly, based on the Talbot formula, the deviation coefficient method is established to realize the flexible control of the $V_{ag}$. Secondly, the wet mixing test is developed to determine the best molding parameters, and a standardized test process is formed. Then, the quantitative relationship model between λ and $V_{ag}$ is established. Moreover, the reduction coefficient is determined by the CT three-dimensional reconstruction method, and the correction basis for the conversion of the wet mixing test results to the asphalt mixture parameters is clarified. Finally, based on the above results, the MaFF method is systematically modified to form a complete skeleton design method.

(1) The conventional gradation design based on the Talbot method is constrained by the n value range of 0.3–0.7, which limits the achievable range of $V_{ag}$. By proposing the deviation coefficient method with reference to the maximum density curve at n = 0.45, this study demonstrates that $V_{ag}$ can be flexibly adjusted through targeted control of the 4.75 mm sieve passing rate. This provides a rational approach for discontinuous gradation design beyond the empirical n value boundaries.
(2) The wet mixing test overcomes this limitation, enabling evaluation of the complete aggregate skeleton. Through the systematic optimization of the molding parameters (2% asphalt content, layered loading, one-sided tamping, and 75 blows), a new detection approach is established that more faithfully simulates the actual compaction behavior of the aggregate.
(3) The strong linear correlation between λ and $V_{ag}$ (R^2^ = 0.996) confirms that the deviation coefficient method offers a predictable and controllable means of tuning the aggregate packing density. More importantly, this relationship reveals a design principle wherein increasing λ systematically enlarges $V_{ag}$, providing a clear theoretical basis for skeleton design optimization.
(4) The comparison between the wet mixing test results and the CT scanning 3D reconstruction reveals a systematic overestimation of $V_{ag}$ by the wet mixing method, with the ratio of the 3D reconstruction values to the wet test values averaging 0.84. This discrepancy is attributed to the inherent differences between laboratory packing and actual mixture compaction. The wet mixing test thus provides a practical laboratory approximation, while the reduction coefficient serves to bridge the gap between laboratory evaluation and actual engineering applications.
(5) This study establishes a standardized framework specifically for the skeleton design component within the MaFF method. This framework provides a systematic alternative to empirical trial-and-error approaches, enabling rational and quantifiable control over aggregate packing in skeleton design.

It should be noted that all experimental data in this study were obtained from a single material system (diabase aggregate, high-viscosity asphalt, 13.2 mm nominal maximum aggregate size) under laboratory conditions, and the empirical relationship between λ and $V_{ag}$ has not been cross-validated by an independent dataset. Nevertheless, the proposed method demonstrates a feasible and effective approach for quantitatively evaluating aggregate packing and guiding skeleton design within the tested material system. Its applicability to a broader range of aggregate types, asphalt binders, gradations, or field conditions requires further systematic validation.

## Figures and Tables

**Table 1:** The basic performance of high-viscosity modified asphalt.

Technical Index	Unit	Standard	Test Result	Test Method
Penetration (25 °C, 100 g, 5 s)	0.1 mm	30~60	42	T 0604-2011
Softening point	℃	≥80	95	T 0606-2011
Ductility (5 °C)	cm	≥20	37	T 0605-2011
Rotary viscosity (135 °C)	Pa·s	≤3	2.8	T 0625-2011
Dynamic viscosity (60 °C)	Pa·s	≥400,000	670,000	T 0620-2025

**Table 2:** Technical indexes of aggregate and filler.

Material	Technical Index	Unit	Standard	Test Result	Test Method
Coarse aggregate	Stone crushing value	%	≤26	18.3	T 0316-2005
	Los Angeles abrasion	%	≤28	17.5	T 0317-2005
	Water content	%	≤2	0.91	T 0305-1994
	Apparent relative density	/	≥2.6	2.87	T 0309-2005
Fine aggregate	Apparent relative density	/	≥2.5	2.82	T 0328-2005
	Sand equivalent	%	≥60	73.6	T 0334-2005
	Mud content	%	≤3.0	0.9	T 0333-2005
	Robustness	%	≤12	4.3	T 0340-2005
Filler	Apparent density	g/cm^3^	≥2.50	2.82	T 0352-2000
	Water content	%	≤1.0	0.3	T 0332-1994
	Hydrophilic coefficient	/	<1	<1	T 0353-2000

**Table 3:** Passing percentage of aggregate fractions based on Talbot formula with n=0.45 (%).

Particle size (mm)	13.2	9.5	4.75	2.36	1.18	0.6	0.3	0.15	0.075	<0.075
Percent of pass (%)	100.00	86.24	63.13	46.08	33.74	24.88	18.22	13.33	9.76	0.00

**Table 4:** Passing percentage of aggregate fractions by deviation coefficient method (%).

*λ*	Particle Size (mm)								
	13.2	9.5	4.75	2.36	1.18	0.6	0.3	0.15	0.075
0	100	86.24	63.13	46.08	33.74	24.88	18.22	13.33	9.76
0.1	100	84.25	57.80	42.45	31.34	23.37	17.37	12.98	9.76
0.2	100	82.26	52.46	38.82	28.94	21.86	16.52	12.62	9.76
0.3	100	80.27	47.12	35.19	26.54	20.35	15.68	12.26	9.76
0.4	100	78.27	41.78	31.56	24.15	18.83	14.83	11.91	9.76
0.5	100	76.28	36.45	27.92	21.75	17.32	13.99	11.55	9.76
0.6	100	74.29	31.11	24.29	19.35	15.81	13.14	11.19	9.76
0.7	100	72.30	25.77	20.66	16.95	14.30	12.30	10.83	9.76
0.8	100	70.31	20.44	17.03	14.56	12.79	11.45	10.48	9.76
0.9	100	68.32	15.10	13.39	12.16	11.27	10.61	10.12	9.76
1.0	100	100.00	9.76	9.76	9.76	9.76	9.76	9.76	9.76

**Table 5:** The test results of the wet mixing test and its corresponding coefficient of variation.

Index		$V_{ag}$ (%)			Coefficient of Variation (%)
		Value	Average	Standard Deviation	
Asphalt–aggregate ratio (%)	1	32.82	32.51	0.266	0.82
		32.54			
		32.17			
	2	31.69	31.25	0.381	1.22
		31.30			
		30.76			
	3	33.01	32.48	0.393	1.21
		32.36			
		32.07			
	4	32.76	32.37	0.278	0.86
		32.13			
		32.22			
Number of tamping strokes (Strokes)	0	34.28	33.24	0.795	2.39
		33.09			
		32.35			
	1	31.86	31.25	0.452	1.45
		31.11			
		30.78			
	2	31.92	31.10	0.600	1.93
		30.88			
		30.50			
Number of blows (Blows)	50	33.16	32.54	0.440	1.35
		32.27			
		32.19			
	75	32.13	31.25	0.624	2.00
		30.75			
		30.87			
	100	32.13	31.6	0.386	1.22
		31.22			
		31.45			

**Table 6:** $V_{ag}$ of gradations under different deviation coefficients.

λ	0	0.1	0.2	0.3	0.4	0.5	0.6	0.7	0.8	0.9	1
*V_ag_* (%)	24.13	25.41	26.33	28.24	29.29	31.54	32.69	34.45	36.10	37.12	38.29
σ	0.35	0.81	0.42	0.57	0.22	0.69	0.41	0.80	0.43	0.84	0.91

**Table 7:** Comparison between experimental measured values and prediction intervals.

λ	$V_{ag}$	Scope of Prediction
0	24.13	[23.176, 24.163]
0.1	25.41	[24.751, 25.571]
0.2	26.33	[26.315, 26.990]
0.3	28.24	[27.859, 28.429]
0.4	29.29	[29.371, 29.890]
0.5	31.54	[30.845, 30.845]
0.6	32.69	[32.287, 32.950]
0.7	34.45	[33.707, 34.512]
0.8	36.10	[35.117, 36.086]
0.9	37.12	[36.519, 37.666]
1.0	38.29	[37.903, 39.235]

**Table 8:** Results of CT scanning 3D reconstruction method.

λ	0	0.1	0.3	0.5	0.7
$V_{ag}$ of test (%)	24.13	25.41	28.24	31.54	34.45
$V_{ag}$ of 3D reconstruction (%)	21.51	22.36	24.00	27.12	28.59
Ratio	0.85	0.88	0.85	0.86	0.83

**Table 9:** Bulk specific gravity of aggregate fractions.

Size (mm)	13.2	9.5	4.75	2.36	1.18	0.6	0.3	0.15	0.075
$\gamma_{n}$	2.870	2.815	2.800	2.802	2.811	2.824	2.816	2.804	2.803

## Data Availability

The original contributions presented in this study are included in the article. Further inquiries can be directed to the corresponding author.

## References

[1] Chen X., Wang H. (2024). Hydro-Mechanical Analysis of Water-Induced Pothole Patch Failure in Asphalt Pavement. Constr. Build. Mater..

[2] Li H., Azarijafari H., Kirchain R., Santos J., Khazanovich L. (2024). Surrogate Modelling of Surface Roughness for Asphalt Pavements Using Artificial Neural Networks: A Mechanistic-Empirical Approach. Int. J. Pavement Eng..

[3] Saltan M., Khaliqi M.H. (2024). Effects of Utilization of Rejuvenator in Asphalt Mixtures Containing Recycled Asphalt Pavement at High Ratios. Case Stud. Constr. Mater..

[4] D’Angelo J.A., Baumgardner G. (2026). Evaluation of Asphalt Binder Properties and Polymer Modifications Effect on Asphalt Mixture Cracking. Transp. Res. Rec..

[5] Xue C., Jiang C., Yang R., Li X., Cheng H., Ye J., Yang L., Sun L., He C. (2025). Effect of Asphalt Overlay on Fatigue Characteristics and Fatigue Life of In-Service Asphalt Pavement Mixtures. Constr. Build. Mater..

[6] Guo R., Nian T., Sun J., Yang Y., Lu X., Zhang J. (2025). Ultra-Thin Asphalt Pavement Preservation Layer: Performance Enhancement Mechanism, Key Technologies, and Development Trends. Case Stud. Constr. Mater..

[7] Zhao C., Shi Z., Zhong X. (2021). A Proposal for an Approach for Meso Scale Modeling for Concrete Based on Rigid Body Spring Model. Comput. Concr..

[8] Al-Ammari M., Dong R., Nasser M., Al-Maswari A. (2025). Innovative Machine Learning Approaches for Predicting the Asphalt Content During Marshall Design of Asphalt Mixtures. Materials.

[9] Liu J., Liu F., Wang L. (2024). Acceleration of Superpave Mix Design: Solving Multi-Objective Optimization Problems Using Machine Learning and the Non-Dominated Sorting Genetic Algorithm-II. Transp. Res. Rec..

[10] Hassan H.M.Z., Elkashef M. (2025). Influence of Gradation on Cracking and Rutting Resistance in Asphalt Mixtures Using the Bailey Method. Int. J. Pavement Eng..

[11] Zhou Y., Ma S., Gan C., Wang W., Yu P., Zheng X., Zhang P., Liu B., Zhao H. (2025). The Effect of Air Void on the Laboratory Properties of Polyurethane Mixtures. Coatings.

[12] Li X., Fu J., Yang F., Cao H., Zhang Z., Liu F., Huang J., Li Y. (2024). Laboratory Investigation for the Bridge Deck Pavement Performance of Conventional Asphalt Mixtures Based on Fuzzy Comprehensive Evaluation Method. Case Stud. Constr. Mater..

[13] Zheng Y., Chen S., Huang W., Cai X., Huang J., Wu K. (2023). Principle Analysis of the Mix Design and Performance Evaluation of the Asphalt-Filler Volume Equivalent Substitution Method. Constr. Build. Mater..

[14] Haddad M., Khedaywi T. (2025). Segregation of Asphalt Concrete Mixtures—State of the Art. Civ. Eng. Archit..

[15] Khedaywi T., Haddad M., Hwarie S. (2025). Effect of Waste Iron Powder on Properties of Asphalt Concrete Mixtures: State of the Art. Int. J. Pavement Res. Technol..

[16] Khedaywi T., Haddad M., Al-Migdady A. (2025). Waste Polyethylene Effect on Asphalt Binder and Mixture Properties: State of the Art. Adv. Sci. Technol..

[17] Khedaywi T., Haddad M., Alyaseen S. (2023). Effect of Filler Materials on Marshall Properties and Rutting of Asphalt Mixtures Using Wheel Tracking Test: A Comparative Study. J. Eng. Sci. Technol..

[18] Khedaywi T., Haddad M., Al-Masaeid H., Abu Mharib I., Hawari S. (2026). Characterization of Binder and Asphalt Mixture Modified with Waste Toner. Int. J. Transp. Sci. Technol..

[19] Gaxiola-Hernandez A., Ossa-Lopez A. (2018). Effect of Water Exposure on the Physicochemical Properties of Impervious Asphalt Concrete. J. Mater. Civ. Eng..

[20] Król J.B., Khan R., Collop A.C. (2018). The Study of the Effect of Internal Structure on Permeability of Porous Asphalt. Road Mater. Pavement Des..

[21] Wang H., Wang C., You Z., Yang X., Huang Z. (2018). Characterising the Asphalt Concrete Fracture Performance from X-Ray CT Imaging and Finite Element Modelling. Int. J. Pavement Eng..

[22] Wu K., Nie G., Cai X., Xu X., Zheng Y., Huang W., Wang Z., Huang J. (2024). Research on Theory and Design Method of Fluid Mastic Asphalt Mixture(FMA). China J. Highw. Transp..

[23] Peng T., Zheng Y., Wu K., Cai X., Huang W., Huang J., Zhang H. (2026). Experimental Characterisation and Modified Burgers Modelling of Viscoelastic Creep Behaviour in a Fluid Mastic Asphalt Mixture (FMA). Int. J. Pavement Eng..

[24] Chen Z., Huang W., Wu K., Nie G., Zheng Y., Song J., Cai X., Wu J., Huang J. (2023). Performance Degradation of Styrene-Butadiene-Styrene Modified Asphalt Binder at Ultra-High Temperature: Process of Construction in Fluid Mastic Asphalt. Case Stud. Constr. Mater..

[25] Chu X., Wu K., Cai X., Huang W., Huang J., Zheng Y., Peng T., Zhang H., Lao C. (2025). Study on the Long-Term High-Temperature Stability of the Fluid Mastic Asphalt Mixture Based on Rutting Tests. Constr. Build. Mater..

[26] Nie G., Wu K., Cai X., Huang W., Huang J., Zhou D., Zheng Y. (2025). Investigation of the Design and Performance of a Fluid Mastic Asphalt Mixture. Int. J. Pavement Eng..

[27] Zheng Y., Sun J., Peng T., Wu K., Huang W., Cai X., Chu X., Lao C., Huang J. (2026). Crack Resistance and Self-Healing Evaluation of Fluid Mastic Asphalt (FMA) Mixtures with Wide-Range Asphalt Content Design. Road Mater. Pavement Des..

[28] Zheng Y., Mi M., Wu K., Song J., Huang W., Cai X., Chu X., Lao C., Nie G., Huang J. (2025). Mix Design and Comprehensive Road Performance Assessment of Fluid Mastic Asphalt. Case Stud. Constr. Mater..

[29] Song J., Wu K., Chen B., Cai X., Zha Z. (2026). Effects of Filling Degree on Skid Resistance in Fluid Mastic Asphalt Mixtures. Road Mater. Pavement Des..

[30] Xu K., Song J., Li Z., Fang G., Zha Z., Wu K. (2025). Research and Application of Rock Asphalt Dry-Modified Fluid Mastic Asphalt Mixture for Reflective Crack Treatment in Asphalt Pavements. Case Stud. Constr. Mater..

[31] Duan S., Wu X., Wang H., Hu J., Luan Y., Ma T. (2024). Design and Evaluation of Semi Self-Compacting Cold Mix Polyurethane Mixture for Steel Bridge Deck Pavement. Constr. Build. Mater..

[32] Xiong R., Jiang W., Yang F., Li K., Guan B., Zhao H. (2019). Investigation of Voids Characteristics in an Asphalt Mixture Exposed to Salt Erosion Based on CT Images. Materials.

[33] Huang W., Fan L., Peng S., Qi Y., Chen G., Liu X. (2026). Experimental Study on the Principal Factors Influencing the Effectiveness of Microbial-Induced Calcium Carbonate Precipitation in Asphalt Concrete Crack Repair Based on Response Surface Methodology. Road Mater. Pavement Des..

[34] Cui W., Wu K., Cai X., Tang H., Huang W. (2020). Optimizing Gradation Design for Ultra-Thin Wearing Course Asphalt. Materials.

[35] Zhong C., Qian G., Gong X., Yu H., Jun C., Zhong Y., Ma J., Gu H. (2025). Study on Mesostructure Evolution Behavior of Asphalt Mixture Compaction Process Based on Deep Learning Image Processing. Constr. Build. Mater..

[36] Jing H., Dan H., Liu X., Ma S., Wang Z. (2025). Multiphysics Coupling and Mesoscopic Seepage Characteristics of Porous Asphalt Pavement Based on a Three-Dimensional Heterogeneous Pore Structure. Constr. Build. Mater..

